# Assessment of Pesticide-Related Pollution and Occupational Health of Vegetable Farmers in Benguet Province, Philippines

**DOI:** 10.5696/2156-9614-7.16.49

**Published:** 2017-12-18

**Authors:** Jinky Leilanie Lu

**Affiliations:** 1 National Institutes of Health, University of the Philippines Manila, Republic of the Philippines; 2 College of Arts and Sciences, University of the Philippines Manila, Republic of the Philippines

**Keywords:** vegetable farmers, pesticide exposure, pesticide pollution, occupational health

## Abstract

**Background.:**

Agriculture accounts for 20% of the national income in the Philippines. In order to boost agricultural activity and prevent crop damage, farmers rely on pesticides for vector control and management.

**Objectives.:**

The present study assessed the pesticide exposure and occupational health of agricultural farmers in the Philippines. The study site is one of the largest vegetable-producing provinces in the Philippines.

**Methods.:**

This study employed both a survey questionnaire and physical health assessment, including a mental state examination. Pesticide exposure was estimated based on the duration of pesticide use, as well as the amount per spray application. The data results were segregated by gender, as women are also heavily engaged in agriculture in this part of the Philippines.

**Results.:**

The results showed that pesticide exposure usually occurred during agricultural activities such as spray applications in the field (63.7%), mixing (38.4%), loading (34.1%) and field re-entry (9.7%). The most frequently used pesticides were Tamaron, Selecron, and Dithane. The mean duration of pesticide exposure was 14.23 years for males and 15.4 years for females. The longest used pesticide among males was Sumicidine (16.2 years), and Tamaron for females (18 years). In terms of amount used, the average was 147 ml per spray application for males and 65.5 ml for females. Exposure to pesticides was expressed in number of years and amount used per spray application, and the average exposure of males was 2,024.43 ml/years and 993.55 ml/years for females. Among farmers, 49% complained of being sick due to their work. Of those who became ill, a large percentage (69.8%) did not receive any medical attention. The most prevalent health symptoms were muscle pains (63.3%), muscle weakness (55%), and easy fatigability (52.4%). For the mini-mental state examination, abnormalities were found in 5.4% of males and 13.3% of females. The use of insecticides was associated with weakness, easy fatigability and weight loss.

**Discussion.:**

The present study demonstrated frequent and significant duration of pesticide use among farmers in Benguet province, Philippines.

**Conclusions.:**

Pesticide exposure was considerable among the farmers in the present study. The occupational health conditions reported by the study subjects can be linked to their pesticide use. Although this study assessed risk factors associated with general health symptoms, further investigation is needed to determine specific pesticide exposure-health correlations.

**Participant Consent.:**

Obtained

**Ethics Approval.:**

The study was approved by the Research Ethics Board of the University of the Philippines, Manila, which is recognized and accredited by the Forum for Ethical Review Committees in Asia and the Western Pacific (FERCAP).

## Introduction

Agriculture is a vital industry in the Philippines, accounting for 20% of the national income. In order to boost agricultural activity and prevent crop damage, farmers rely on pesticides for vector control and management.^1^ A study carried out in thirty irrigated rice areas in the Philippines found that most farmers applied chemicals to manage golden apple snails and weeds that damaged or competed with rice.^2^ However, farmers and farm workers usually handle pesticides without following label instructions, and are involved in loading, mixing or spraying the pesticides without the use of personal protective equipment. Disparities also exist between perceptions of agricultural productivity and safe use of pesticides.^3^

The aim of the present study was to assess the pesticide exposure and practices of farmers, as well as determine their physical health condition. This study was conducted in Benguet, which is the largest vegetable-producing area in the northern Philippines. Planting and harvesting occur year-round in this area. Based on the data from the Philippines Statistics Authority, the major source of employment for most of the people in Benguet is agriculture. At least 54% of the labor force is engaged in vegetable or cut flower farming. As of 2010, Benguet had a total land area of 2,833 square kilometers, the majority of which is planted with crops.^4^ The results of the present study can be used to strengthen the Philippine's surveillance programs on pesticides and health hazard mitigation efforts.

## Methods

Farmers from the various municipalities in Benguet were the target population. The subjects consisted of five hundred and thirty-four farmers selected through cluster sampling. As the size of the target population was unknown due to data unavailability, the following sample size equation was used.

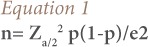
Where n is sample size, Z is reliability level or confidence level, p is true proportion of success, and e is margin of error.

The structured survey questionnaire included items on socio-demographic profiles, medical history, and pesticide use, including history, types of pesticides, duration, amount and frequency of use, perceived adverse health symptoms related to pesticide exposure, and pesticide health and safety practices including use of personal protective equipment, administrative controls, and engineering measures. The questionnaire can be found in Supplemental Material.

Cluster sampling is a sampling technique where the entire population is divided into clusters and a random sample of these clusters is selected. Since the study assumed that pesticide use was similar in all six municipalities, the sample variation within each cluster was assumed to be negligible. Data collection from the target population consisted of structured personal interviews, physical and neurological examinations, and blood sampling.

A nine-page survey questionnaire was developed. The questionnaire included questions on demographics, past and present medical history, family medical history, obstetric-gynecological history for females, pesticide use and practices, and health effects.

Exposure assessment was performed using the type, amount, duration and frequency of reported pesticide use for farmers' agricultural crops. This was complemented by a physical health assessment.

Pesticide exposure was captured in this study through a survey questionnaire, similar to that used by Povey et al, where a job exposure matrix was created using self-reported information and questionnaire.^5^

Both self-reported pesticide use and health assessments were used to examine the relationship between pesticide exposure and adverse health symptoms. Cumulative risk exposure was also measured using duration and number of years of pesticide usage. Similarly, a hazard index and spraying journal were used to calculate pesticide risk in a study by Larsson et al.^6^

A physical health assessment was conducted among the 534 farmers to validate their self-reported health symptoms in the survey questionnaire based on symptoms provided by the National Pesticide Control and Management Center (NPCMC) and World Health Organization (WHO).^7^,^8^ The Mini-Mental State Examination (MMSE) or Folstein test^9^ was also administered, which is a 30-point questionnaire covering several areas of cognition used to estimate the progression of cognitive impairment or to track the cognitive development of individuals. A score of 26 and above is considered to be within normal limits, and a score of 23 and below is considered to be abnormal.^9^

Medical doctors administered the examination. Broken down by cognition areas, the test is composed of a total possible score of 10 for orientation, 3 for instant recall, 5 for calculation, 3 for recent memory, 1 for phrase repetition, 2 for anomia, 1 for reading comprehension, 3 for 3-step commands, 1 for sentence construction, and 1 for object construction. This yields a total possible score of 30.

The health and work practices data, including the physical health examinations, were analyzed using the Statistical Package for the Social Sciences (SPSS) program (version 24.0, SPSS Inc, Chicago, Illinois). Both descriptive and inferential statistical methods were used. Descriptive statistics included frequency and percentage distribution, and mean and standard deviation. Inferential statistics included chi-square analysis.

Participants were informed about the nature of the study including the research objectives, purposes and goals. They were also informed about the blood extraction procedure, its purposes and risks, and participants gave written informed consent. Participants were also assured of the confidentiality of study data. The study was approved by the Research Ethics Board of the University of the Philippines, Manila, which is recognized and accredited by the Forum for Ethical Review Committees in Asia and the Western Pacific (FERCAP).

## Results

Out of the 534 respondents, 53.4% were male and 46.6% were female. The majority were married (80.5%) and the mean age was 47 years. Almost half (47.9%) were employed as agricultural workers, 23.7% as agricultural pesticide applicators/mixers or loaders, and 12% as growers. Approximately 20% of households had children aged 13–15 years old, and 16.6% and 16.2% had children aged 10–12 and 7–9 years old, respectively. A total of 94.5% of respondents had lived at their present address for more than five years and 38.7% said their houses were situated 1 to 50 meters from the agricultural fields. More than half (55.5%) of household members were working directly with pesticides. A number of households (15.9%) had up to three younger children (under 15 years of age) directly exposed to pesticides.

The duration and frequency of pesticide exposure are important risk factors for the development of chronic and long-term health effects. In the study area in Benguet, the most commonly used pesticides were Tamaron, followed by Dithane (mancozeb) and Sumicidine (fenvalerate) The active ingredient in Tamaron is methamidophos and it is classified as an organophosphate pesticide. Dithane, a mancozeb, is a dithiocarbamate pesticide, and Sumicidine is a pyrethroid with an active ingredient of fenvalerate. In addition, Selecron is composed mainly of prochloraz-Mn and spinosad. By gender, Tamaron was the most frequently used pesticide by males (39.3%) compared to 32.9% of females. Among females, Dithane was the most commonly used pesticide (35.7%), compared to 32.3% among males. The gender distribution of use of various types of pesticides is shown in Table 1.

**Table 1 —:**
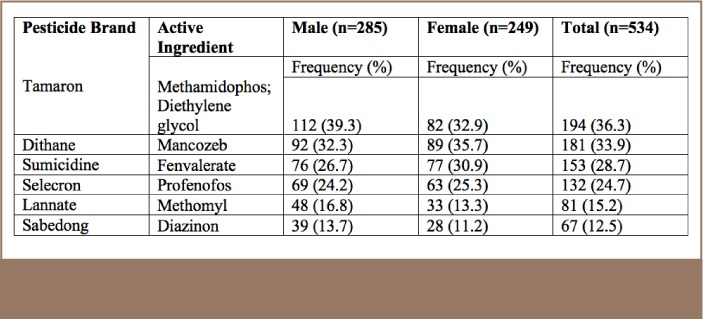
Frequency and Percentage Distribution of Commonly Used Pesticidesby Gender (n=534)

Pesticide exposure usually occurred during agricultural activities such as spray application in the field (63.7%), mixing (38.4%), loading (34.1%) and field re-entry (9.7%). Totals and gender specific distribution of activities at the time of exposure are shown in Table 2.

**Table 2 —:**
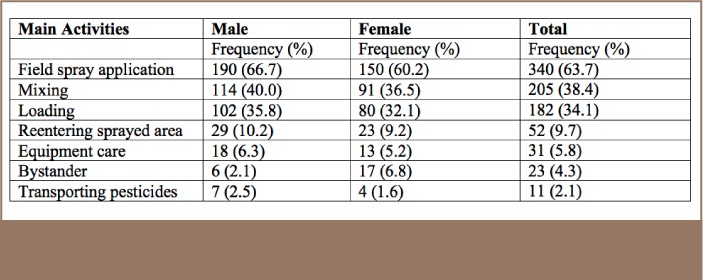
Frequency and Percentage Distribution of Occupational Activities at Time of Exposure (n=534)

Table 3 shows that the mean duration of pesticide use for males was 14.23 years, and 15.4 years for females. The longest used pesticide among males was Sumicidine at 16.2 years, and Tamaron for females at 18 years. In terms of amount used, the male average was 147 ml per spray application and 65.5 ml for females. The highest reported pesticide application for males and females was Sabedong, at 360 ml and 83 ml per spray application, respectively. Sabedong is known to cause headache, blurred vision, nervousness, salivation, sweating, chest discomfort, and miosis, while Sumicidine has been associated with excessive sweating, blurred vision, and headache.^10^

**Table 3 —:**
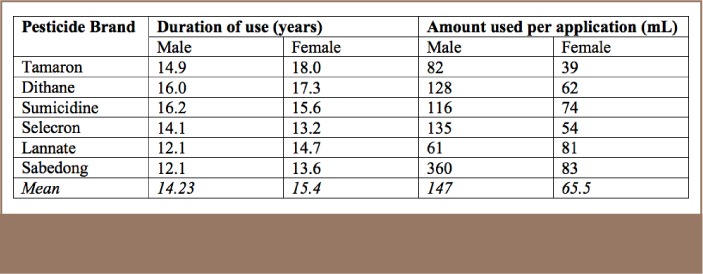
Duration and Amount of Pesticide Use Among Farmers by Gender

In terms of exposure to pesticides, expressed in terms of number of years and amount used per spray application, Table 4 shows that the average exposure of males to the most commonly used pesticides was 2024.43 ml/year and 993.55 ml/year for females. The highest exposure for males was for Sabedong at 4,356 ml/year, and Sumicidine for females at 1154.4 ml/year. Sabedong has an active ingredient of cypermethrin and is a pyrethroid type of pesticide. Sumicidine has an active ingredient of fenvalerate and is also a pyrethroid type of pesticide (*Table 4*).

**Table 4 —:**
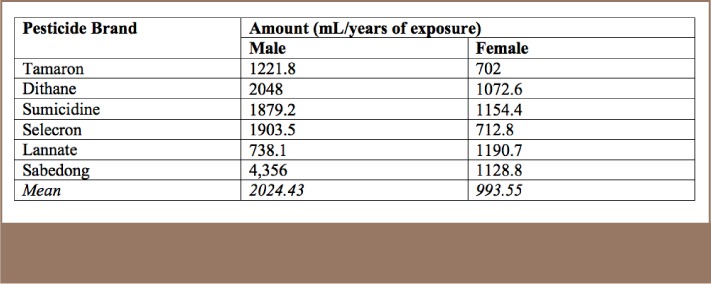
Pesticide Exposure Among Farmers by Gender

Forty-nine percent (49%) of farmers complained of being sick due to their work. Of those who got ill, a large percentage, 69.8%, did not receive any medical attention and only 24.3% received some medical attention. Occupational exposure was more prevalent (84.8%) than accidental exposure (15.2%).

With regard to the general symptoms reported by the farmers, the most prevalent were muscle pains (63.3%), followed by muscle weakness (55%), and easy fatigability (52.4%). Among males and females, the most frequent health problems were muscle pain, muscle weakness, and easy fatigability (*Table 5*).

**Table 5 —:**
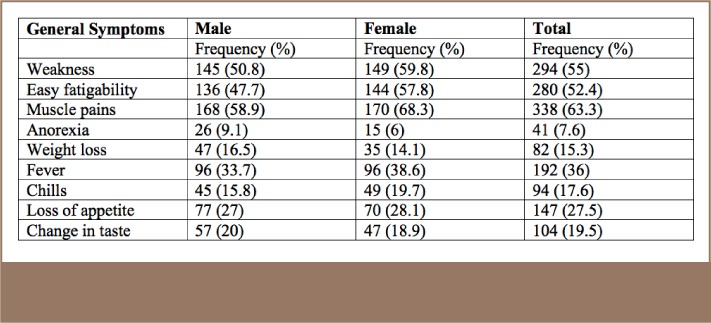
General Health Symptoms Reported by Subjects by Gender

The most common eye problem was blurring of vision, followed by eye pain, and eye tearing. These were also the most prevalent eye symptoms reported by both males and females (*Table 6*).

**Table 6 —:**
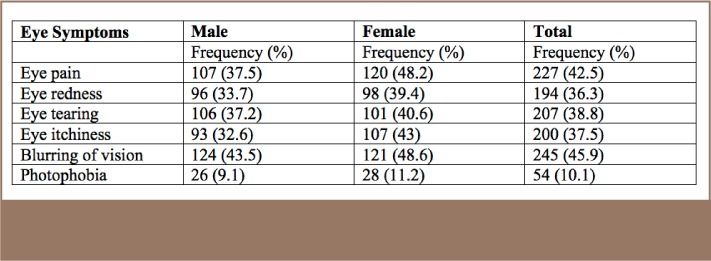
Eye Symptoms Reported by Subjects by Gender

For neurologic symptoms, the most prevalent were headaches and dizziness, followed by vertigo, for both males and females (*Table 7*).

**Table 7 —:**
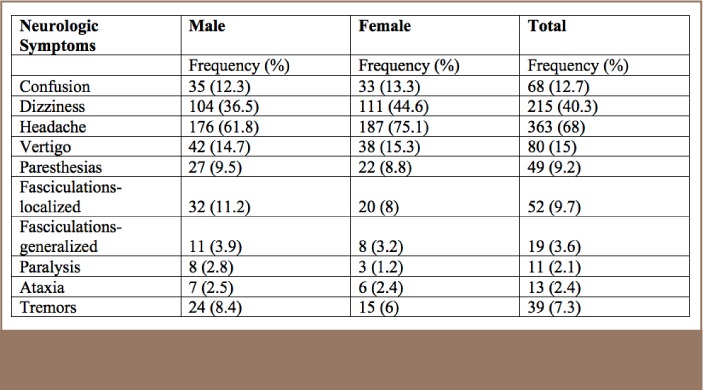
Self-Reported Neurologic Symptoms by Gender

For the mini-mental state examination results, abnormalities were found in 5.4% of males and 13.3% of females (*Table 8*). The mean scores are shown in Table 9. The mini-mental state examination is also called the Folstein test, and is a 30-point questionnaire covering several areas of cognition. It is a commonly used tool in medicine, public health and allied medicine as it is simple to administer.^11^ It can also be used to estimate the progression of cognitive impairment or to track the cognitive development of an individual person.^9^

**Table 8 —:**
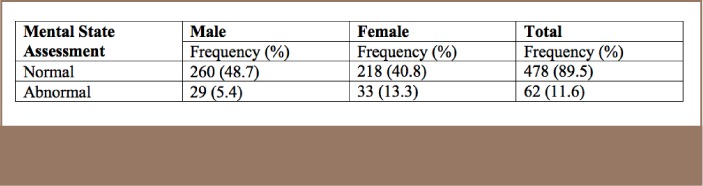
Mental State Examination Scores

**Table 9 —:**
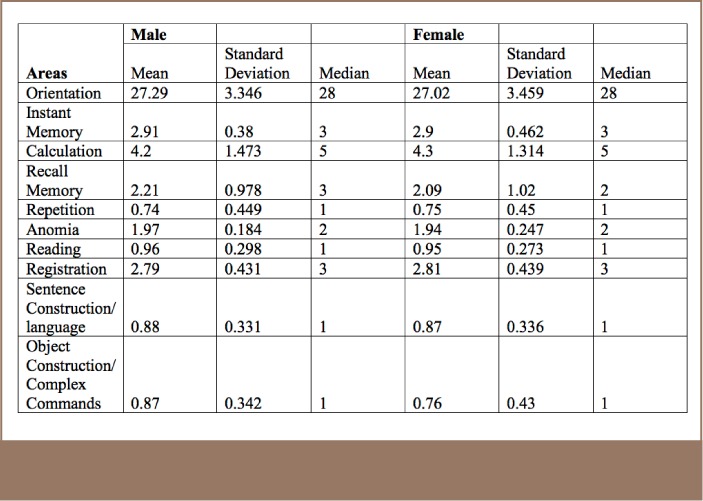
Mental State Examination Scores by Cognition Area

## Discussion

The present study found that both males and females were engaged in farming and agricultural activities. Among the study households, 15.9% reported having children under 15 years of age who were exposed to pesticides. A systematic review conducted by Hyland et al. using biological samples and environmental monitoring found that children of farmworkers were more at risk of pesticide exposure at home than children of non-agricultural farmers.^12^ This review demonstrated the possible adverse health risks among children of farmworkers. A study by Van Maele-Fabry et al. also found a positive relationship between residential pesticides and the development of brain tumors among children, and recommends limiting the use of pesticides and the creation of public policy concerning pesticide use.^13^

The study site is also a farming community with households in vicinity to the farms. Farming communities in rural areas can be exposed through environmental drift and volatilization of agricultural pesticides, especially in closely linked farm-residential areas. A previous study found that due to the environmental drift of pesticides, there was an increased risk of Parkinson's disease.^14^ Proximity to agriculturally-sprayed areas was used as a proxy for pesticide exposure among children, and this was validated by Bukalasa et al.^15^ in a study using the Dutch birth cohort study. The authors used spatial data on the presence of crops cultivated within 50, 100, 500 and 1000 m of the study homes to calculate the surface area of specific crops that were applied with pesticides. The results showed that pesticides with known irritant properties to the respiratory system were potentially applied within these distances. The study also used combined acreage of fields based on farmer-reported pesticide use as basis for the determination of pesticide exposure.

The results of the present study demonstrated frequent and significant duration of pesticide use among farmers. Frequently used pesticides included Tamaron, Dithane, Sumicidine, Selecron, and Lannate. Tamaron is of a specific interest as it was the most frequently used pesticide. Its active ingredient is methamidophos and it is classified as an organophosphate pesticide which may cause abnormalities in blood cholinesterase activity. The danger of pesticide contamination usually increases with the dosage (concentration) and the speed of treatment. For example in pesticide poisoning, the ‘golden hour’ is the period within which medical management must be given to prevent a generalized and potentially fatal effect.^16^

Tamaron is composed of organophosphorus esters. Organophosphorus terminates the action of acetylcholine neurotransmitters, producing delayed and irreversible neuromuscular effects usually seen in the extremities. The number of years the farmers reported using pesticides was notable, 14.9 years for males using an average of 82 ml of pesticides per application, and 18 years for females using an average of 39 ml per application. This is in contrary to the findings of Zadjali et al. which revealed a decreased usage of pesticides among older farmers.^17^

In terms of toxicity class, Tamaron poses the greatest risk as its toxicity rating is Class 1, which indicates extreme toxicity, followed by Selecron and Lannate with a toxicity class rating of 2. The other pesticides have a toxicity rating of 4, which is the least toxic. Tamaron, a highly toxic pesticide, was used by farmers in the largest amount in terms of ml/year, posing a risk to their health (*Table 10*).

**Table 10 —:**
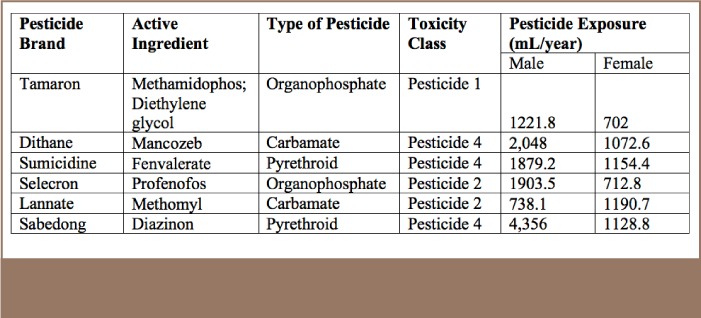
Active Ingredients, Type of Pesticide, Toxicity Rating, and Pesticide Exposure

As shown in Table 2, occupational exposure to pesticides was examined. This is similar to a previous study where a job occupational exposure matrix was developed using the categories of work history and job description, among others, in order to examine the exposure estimates for polychlorinated biphenyls.^18^ The possibility of recall bias was reduced in this study due to the fact that farmers used pesticides daily, and they regularly purchased them in the market. Their daily lives revolved around farming and agriculture, and pesticide use was a major concern as it affected their costs and income in terms of agricultural productivity.

In a study by Benke et al.,^19^ a job exposure matrix was performed to assign cumulative exposure to workers. Where quantitative data were available, cumulative exposures were calculated for all workers performing the same job, expressed as mg m−3×years. In the present study among farmers in the Philippines, the amount and duration of pesticide use were also computed. Based on the amount and duration of pesticide usage, it was found that the mean ml/years of pesticide usage was 2024.4 ml/yr for male farmers and 993.5 ml/yr for females.

In the present study of Benguet area farmers, several adverse health symptoms were reported such as muscle pain, weakness and easy fatigability. The neurodevelopment of an individual can affect their neuropsychological functioning and may lead to suicidal ideation. In a study in South Korea among 1958 male farm workers, an association was found between pesticide poisoning and suicidal ideation, the latter being defined as a thought of harming oneself or trying to take one's own life.^10^ In that study, there was a 2.48-fold increase in risk with multiple pesticide poisoning among male Korean farmers. Increased risk of mental depression was found among those who reported using pesticides in a population of 2151 Koreans, particularly those who reported pesticide use longer than 20 years (odds ratio, 2.35; 95% confidence interval, 1.41–3.88). Experimental studies have shown that pesticides act as neurotoxic and endocrine-disrupting toxins.^20^ A study in China found that a 10% increase in pesticide use in rice increased a key medical disability index by 1% among rural residents 65 and older, resulting in $2.13 and $0.64 million dollars in medical and family care costs, respectively.^21^

There are a number of short-term and long-term adverse health symptoms associated with pesticide exposure. Short-term symptoms include skin and eye irritation, headaches, dizziness, and nausea, while long-term effects include cancer, asthma, and diabetes.^10^ The present study found a significant duration and level of pesticide exposure among farmers in Benguet, the Philippines.

Eye symptoms were commonly reported among Benguet farmers, including blurring of vision, eye pain, and tearing. Similarly, eye irritation, particularly toxicity to the cornea, has been documented to be caused by pesticides,^22^ resulting in ocular irritation and damage to normal vision. Additionally, chronic exposure to pesticides is associated with eye pathologies such as keratectasia and corneal neovascularization, disorders that could eventually lead to diminished vision and blindness if not treated.

The effect of pesticides on neurologic functioning was also investigated in a study by Bahrami et al.,^23^ which determined that the combined effect of nicotine and pesticides among tobacco farmworkers predisposed them to neurological changes in the brain. At the molecular level, the study found that despite having the same functional connectivity density and strength, brain networks in farmworkers had more clustered and modular structures relative to non-farmworkers. This suggests that chronic exposure to cholinesterase-inhibiting pesticides such as organophosphates and carbamates, in combination with nicotine, can lead to neurological dysfunction. In addition, exposure to organophosphate and carbamate pesticides have been implicated in affecting cholinesterase activity that, in turn, could affect memory.^24^ In the present study, exposure to organophosphates and carbamates may be linked to abnormalities in farmers' mental state examination results. The percentage of abnormalities in the mental status examinations was 5.4% among males and 13.3% among females.

In addition, several risk factors were identified in association with health symptoms experienced by farmers in the present study. The use of insecticides was associated with weakness, easy fatigability and weight loss and use of damaged backpack sprayers was associated with weakness and easy fatigability (*Table 11*).

**Table 11 —:**
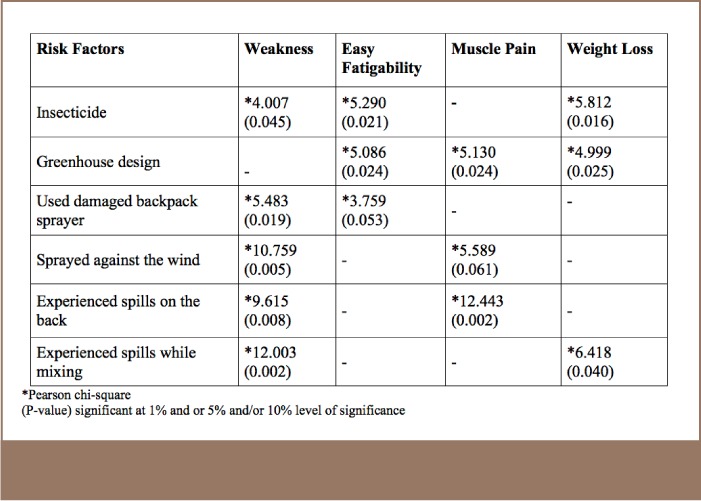
Statistical Association Between Risk Factors and Health Symptoms Among Study Subjects

There are several limitations to the present study. Further studies are needed to conduct biomonitoring such as pesticide metabolites in biologic samples, personal sampling of pesticide residues on the skin, or vapor inhalation within the breathing zone of farmers. A revised job exposure matrix should include a more detailed observation and analysis of the various tasks performed in farming, estimating the corresponding pesticide exposures.

## Conclusions

The present study explored pesticide exposure among farmers in Benguet, the Philippines, as well as possible health symptoms. These exposures suggest that the surrounding community may also be affected by pesticide exposure, with children in particular being at increased risk of exposure. Occupational exposure was the main route of pesticide exposure, mainly during spray application in the fields. The amount and duration of pesticide use were significant among farmers, and farmers manifested health symptoms that have been linked to pesticide exposures in previous studies. It is recommended that the information gathered in this study be used to improve current policies and standards with regard to the surveillance of pesticide use. Further studies should investigate the specific mechanisms of the possible cause-effect relationship between exposure and outcome and adjust for potentially confounding variables such as farm size, amount of pesticide used per application, season, and availability of farm extension services.
