# Supplementary material for: Assessment of Pesticide-Related Pollution and Occupational Health of Vegetable Farmers in Benguet Province, Philippines
Source: J Health Pollut. 2017 Dec 18;7(16):49–57. doi: 10.5696/2156-9614-7.16.49 (PMC6221443; doi:10.5696/2156-9614-7.16.49)
Supplement: Supplementary file 1 [file JHP-2017Dec-Lu_SuppMaterial.pdf]

ANNEX: QUESTIONNAIRE AND PHYSICAL HEALTH ASSESSMENT CHECKLIST GUIDE

GENERAL DATA

Name: First ____________ Last ___________ Age:_____ Sex: M F

Marital Status: ☐single ☐married ☐widow/er ☐separated/ divorced ☐unknown

Barangay:_________________

Educational Attainment: (State number of years in school)

☐grade school______________ ☐high school______________

☐college____________________ ☐vocational_______________

☐post-graduate______________

Occupation: ___ Agricultural worker

___ Agricultural pesticide applicator/mixer/loader

___ Grower

___ Pesticide distributor

___ Housewife

___ Vector control officer

___ Student

___ Other job: ____________________________

HOUSEHOLD

How many people live in your home (other than yourself)? ____ persons

Of these people, how many are children less than 18 years old? ____ children (ages: ____, ____, ____, ____, ____, ____, ____, ____)

Do any of these people work in agriculture or with pesticides?

___Yes ___No __Unknown

If yes, please state how may are 18 years or older: ___ adults

And how many are 13 to 17 years or older: ___ older children

And how many are 12 or younger: ___ young children

HOME ENVIRONMENT

Home Address:____
Length of stay in present address (number of yrs): __________
Since when resided in the address (yr, 1958 or so) __________
Distance from plantation:_______________m Distance from highway__________m

HEALTH HISTORY

Past History	Illness	Family History (specify member)
☐	Hypertension	☐________________________
☐	Diabetes mellitus	☐________________________
☐	Ischemic Heart Disease	☐________________________
☐	Kidney disease	☐________________________
☐	Asthma	☐________________________
☐	Liver Disease	☐________________________
☐	Tuberculosis	☐________________________
☐	Thyroid Disease (specify disease and meds) _______________________	☐________________________
☐	Allergies (specify______)	☐________________________
☐	Cancers (specify______)	☐________________________
☐	Psychiatric Disease (specify)	☐________________________
☐	Autoimmune Disease (specify)	☐________________________

Are you taking medications at the moment? ☐Yes ☐No
If yes, specify ____

PESTICIDE EXPOSURE

Have you ever worked with or used pesticides? □ Yes □ No

For how many years? _______ Years

Kindly fill up the table below for pesticides you use:

Name of Pesticide	Duration of use/ exposure (years)	Frequency of use (how many times of spraying per week?)	How many cropping seasons of use per year

During spraying time, how frequently are you exposed? Specify how many.

____hours in a day ____days in a week _____weeks per month

____months per cropping season ____ cropping seasons per year

What is the average amount of time used to prepare dilution? _____ Minutes

What is the total spraying time/ load ____Minutes ____Hours

Has anyone given you instructions in how to use pesticides through video, audio cassette, classroom lecture, written material, informal talks or any other methods?

___ Yes ___ No ___ Unknown

While working, were you involved in the following pesticide activities?

Mixing? ___ Yes ___ No ___ Unknown

Loading? ___ Yes ___ No ___ Unknown

Applying? ___ Yes ___ No ___ Unknown

Do you eat while handling pesticides? ___ Yes ___No

When you spray, do you use backpack sprayer? □ Yes □ No

If no, specify: ____

Do you wipe sweat off the face with piece of fabric? ____ Yes ____No ___ Unknown

Do you reenter recently sprayed area? ___ Yes ____No ____ Unknown

Do you spray : against the wind? ____ Yes ____No ___Unknown

in front? ____ Yes ____No ___ Unknown

Have you experienced spills:

On the back? ___ Yes ___ No ___ Unknown

While spraying? ___ Yes ___ No ___ Unknown

While mixing? ___ Yes ___ No ___ Unknown

Do you wash your hands after applying pesticides and before eating? ___Yes __ No

Do you take a bath after applying pesticides? ___Yes ___No

STORAGE AND DISPOSAL OF PESTCIDES

Where do you keep your pesticide for field spraying?(specify) _______________

What do you do with used pesticide container ?

 Burn Bury Destroy and throw with ordinary waste

 Sell Others: (specify): ___________________________________

Where do you clean the sprayer and other devices used for mixing /loading?

 Creek Faucet at home In the field

 River Others: (specify): ___________________________________

Where do you clean the PPE (boots, gloves that you used in handling pesticides)?

 Creek Faucet at home Field with drum of water

 River Others: (specify): ____________________________________

Who washes the clothes that you used while handling pesticides?

 Yourself Wife Laundry woman

 Others: (specify) _________________________________

PESTICIDES AND YOUR HEALTH

During the last 12 months, did you become sick or have any complaints because of your work? ___ Yes ___No ___ Unknown

If yes, how many times? ______

What date did the exposure occur? Day___/ Month___/ Year ____

Was this an accidental exposure? ___ Yes ___No ___ Unknown

Was this an occupational exposure? ___ Yes ___No ___ Unknown

In the last 12 months, have you received any medical attention by a doctor, nurse or midwife due to pesticide exposure? ____Yes ____No ____Unknown

Even for those who did not get sick, but has exposure,
How did the pesticide enter your body?

___ Oral (ingestion) ___ Respiratory (inhalation) ___ Ocular (eye)
___ Dermal (skin) ___ Other (specify): ____________
___ Unknown

What was the pesticide being used for?

___ Insecticide ___ Fungicide ___ Tick control ___ Unknown
___ Herbicide ___ Rodenticide ___ Nematocide
___ Other (specify): _______________________________

What was the main activity at the time of exposure?

___Application in the field ____Equipment care ____Bystander
___Field re-entry ____Transportation ____Others
___Mixing ____Public health/vector control______________
___Loading

Where did this exposure occur?

___Farm/field ___Public area ___Home (rural)
___Garden (rural) ___Storage site ___Greenhouse
___Unknown ___Other (specify)________________

What were your symptoms?

General

☐Weakness ☐Anorexia ☐Fever ☐Loss of appetite
☐Easy fatigability ☐Weight loss ☐Chills ☐Change in taste
☐Muscle pains
☐Others (specify)____

EENT

☐Eye pain ☐Blurring of vision ☐Tinnitus ☐Hoarness
☐Eye redness ☐Photophobia ☐Nasal secretion ☐Neck mass
☐Eye tearing ☐Ear ache ☐Nose bleed
☐Eye itchiness ☐Deafness ☐Nasal congestion
☐Others (specify)____

Neurologic

☐Confusion	☐Fasciculations (local)	☐Ataxia
☐Dizziness	☐Fasciculations (general)	☐Hallucinations
☐Headache	☐Convulsions	☐Drowsiness
☐Vertigo	☐Loss of consciousness	☐Tremors
☐Paresthesias	☐Paralysis	

☐Others (specify)____

Gastrointestinal

☐Abdominal pain	☐Hematemesis
☐Nausea	☐Perforation
☐Vomiting	☐Others (specify)____________________
☐Salivation	
☐Throat irritation	
☐GI burn	
☐Difficulty in swallowing	

Respiratory system

☐Coughing	☐Pain on deep breathing
☐Breathlesness	☐Cyanosis
☐Noisy breathing	☐Pulmonary secretions
☐Difficulty of breathing	☐Others (specify)______________________

Cardiovascular system

☐Chest pain	☐Pillow orthopnea
☐Palpitations	☐Calf pains
☐Exertional dyspnea	☐Syncope
☐Arrhytmias	☐Bradycardia
☐Tachycardia	☐Others (specify)_______________________

Integuments/Skin

☐Skin discoloration	☐Blisters	☐Sweating
☐Easy bruisability	☐Skin lesions ________	☐Jaundice
☐Skin rashes	☐Alopecia	☐Nail changes
☐Skin itchiness	☐Pallor	

☐Others (specify)____

How long after exposure were the symptoms felt or seen? ____________ (hours)

Did the symptoms occur: ☐immediately after ☐after quite sometime

How long did the symptoms last? ____Minutes or ____Hours or ____days

What was the outcome?

☐ Recovery, no need to seek consultation

☐ Brought to hospital – consultation, but not admitted

☐ Brought to hospital – admitted

☐ Others

Was treatment provided? ___ Yes ___No ___ Unknown

If yes, where were you treated? ___ Hospital ___ Health center

___ Private Clinic ___ Other: (specify) _________________________

I. Mental Status Examination:

1. Orientation	Points	Score
date	1	
day	1	
month	1	
year	1	
season	1	
place of examination	1	
floor	1	
town/city	1	
province	1	
country	1	
2. Instant Recall<br>bola, aso, puno<br>Number of trials:_____	3	
3. Calculation<br>serial subtraction of 7 from 100 (1 pt each)<br>OR<br>spell T-U-L-A-Y backwards<br>Score no. of correctly placed numbers or letters	5	
4. Recent Memory<br>bola, aso, puno	3	
5. Repeat this phrase<br>botika, bituka, butiki	1	
6. Anomia<br>name pen and watch	2	
7. Reading Comprehension<br>read and perform "IPIkIT ANG MATA"	1	
8. 3 - Step Command		
"Ipikit and mata"	1	
"Ilabas and dila"	1	
"Hawakan ng kanang kamay ang kaliwang tenga"	1	
9. Sentence Construction<br>check for presence of subject and predicate	1	

10. Object Construction<br>construction of intersecting pentagons or objects<br>appropriate for age (see below)	1	
**TOTAL**	**30**	

Objects for Construction:

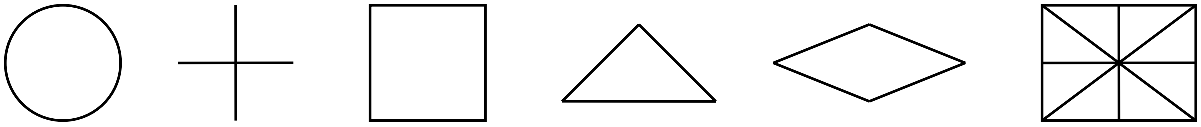

3 yrs old   4 yrs old   4 ½ yrs old   5 yrs old   6 yrs old

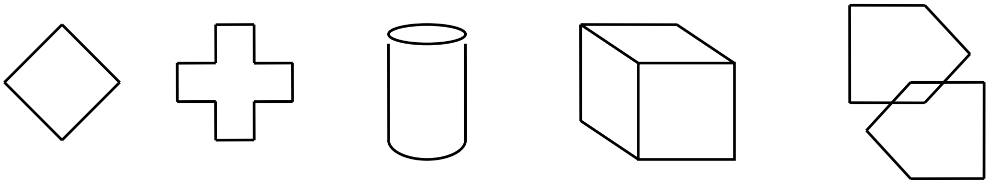

7 yrs old   8 yrs old   9 yrs old   11 yrs old   12 yrs old/above
